# Comparison of Immunostaining with Hematoxylin-Eosin and Special Stains in the Diagnosis of Cutaneous Macular Amyloidosis

**DOI:** 10.7759/cureus.7606

**Published:** 2020-04-09

**Authors:** Fatemeh Sari Aslani, Hadis Kargar, Akbar Safaei, Farideh Jowkar, Motahareh Hosseini, Mozhdeh Sepaskhah

**Affiliations:** 1 Dermatology, Molecular Dermatology Research Center, Shiraz University of Medical Sciences, Shiraz, IRN; 2 Pathology, Shiraz University of Medical Sciences, Shiraz, IRN; 3 Dermatopathology, Molecular Dermatology Research Center, Shiraz University of Medical Sciences, Shiraz, IRN

**Keywords:** amyloidosis, primary cutaneous, crystal violet, immunohistochemistry, diagnosis

## Abstract

Background

Although macular amyloidosis is a relatively rare disease, it is a common cutaneous disease in Asia and the Middle East. On hematoxylin and eosin (H&E) stained slides, early lesions could easily be missed without the use of special stains and/or immunohistochemistry.

Methods

We enrolled 42 patients with the clinical impression of macular amyloidosis who had undergone two 4-mm punch biopsies from 2015 to 2016 at a dermatology clinic affiliated to Shiraz University. Besides, 14 cases with a clinical diagnosis other than macular amyloidosis were selected as the negative control group. Congo red, crystal violet, and immunohistochemical (IHC) staining of CK5 and high molecular weight keratin (HMWK) were performed for each specimen.

Results

H&E slides showed globular depositions in 15 (35.7%) out of 42 patients. None of the patients showed apple-green birefringence with Congo red stain. Evaluation of crystal violet stained sections revealed purplish violet amyloid deposits in 15 (35.7%) patients. IHC study showed expression of CK5 in 52.4% and HMWK in 50% of the patients, which was not a significant difference (p = 0.715). The findings of both IHC markers had a significant difference with H&E stains (p = 0.039) and crystal violet (p = 0.008). Additionally, we found that two punch biopsies from two sites in the involved area did not have a significant preference over one punch biopsy. All of the cases in the control group were negative for amyloid deposition in H&E, special stains, and IHC stained slides as expected.

Conclusions

IHC evaluation using CK5 and HMWK might be a useful tool for diagnosing macular amyloidosis.

## Introduction

Primary cutaneous amyloidosis (PCA) is characterized by the deposition of amyloid in the skin without extracutaneous involvement. Lichen, macular, and nodular are the main variants of amyloidosis, with macular and lichen amyloidoses being more common [1]. Macular amyloidosis (MA) is a relatively rare disease, but it is a common cutaneous disease in Asia, especially in the Middle East [2]. It is characterized by a pruritic reticulated or rippled pattern of symmetrical pigmentation, mostly in the upper back [3]. The pathogenesis of MA is not fully elucidated; however, the amyloid deposit is derived from keratinocytes. Chronic scratching in susceptible individuals is thought to contribute to the mechanism of amyloid deposition [4].

While the diagnosis of MA relies on clinical identification of characteristic skin findings, definitive diagnosis requires histological confirmation [5]. Clinical differential diagnoses are frictional melanosis, notalgia paresthetica, and postinflammatory hyperpigmentation (PIH) [3]. According to several differential diagnoses of pigmented patches on the same anatomical sites with different treatment options, correct diagnosis is necessary for proper and effective treatment. On hematoxylin and eosin (H&E) stain, early lesions contain small, multifaceted, and amorphous globules within the papillae, which are easily missed without the use of special stains and/or immunohistochemical (IHC) staining. Typically, pigmentary incontinence is present without significant epidermal changes. Other diseases with amorphous pink material (e.g., erythropoietic protoporphyria, colloid milium, lipoid proteinosis, and Waldenstrom’s macroglobulinemia) are histologically in the differential diagnosis of MA [5]. The amyloid may be seen with several histochemical stains, including methyl violet, crystal violet, thioflavin T, and Congo red. Congo red is one of the most common staining techniques, as amyloid shows a characteristic apple-green birefringence when viewed under polarized light [6,7]. In MA, false-negative reactions may occur with the Congo red stain, and crystal violet stain is the most useful stain for amyloid keratin [8]. When special stains do not show the presence of amyloid, the ultrastructural study is usually successful in detecting the existence of the protein [5]. IHC studies have shown intense staining of the amyloid with cytokeratin 5 (CK5) antibody and high molecular weight keratin (HMWK) (34betaE12) and have suggested that amyloid is derived primarily from basal keratinocyte [3,9]. Several antikeratin antibodies have been used for detecting amyloid deposition in previous studies, such as CK5, CK6, CK10, CK14, CK17, CK18, CK19, CK5/6/18, CK8/18, CK5/6, CK5/6/8/18, MNF116, HMWK, and AE1/AE3. Among these markers, HMWK and CK5 seem to be more sensitive for amyloid detection [7,10]. The electron microscopic findings included typical filaments of amyloid of 6-10 nm thickness that are straight and non-branching [3].

In this study, we used two punch biopsies from two sites in the involved area in an individual patient with clinical features of MA. Crystal violet and Congo red stain, IHC study using CK5, and HMWK were used to detect amyloid and compare IHC findings with histochemical staining for the diagnosis of MA.

## Materials and methods

Patient selection

Between 2015 and 2016, the archive of a surgical pathology lab in a hospital (Shahid Faghihi) affiliated to Shiraz University of Medical Sciences was searched for cases with the clinical impression of MA who underwent two 4-mm punch biopsies, and 42 cases were selected. Besides, 14 cases with a clinical diagnosis of PIH and old lichen planus were selected as negative controls. H&E slides were reviewed by a dermatopathologist for the evaluation of hyaline bodies in the papillary dermis as a strong clue for the diagnosis of MA.

Crystal violet techniques of staining

 Working crystal violet solution was prepared with the dilution of 10-mL stock crystal violet solution in 300-mL distilled water and 1-mL hydrochloric acid. Then, the staining was performed on paraffin sections using the following method: (1) An 8-µm section of the proper paraffin block was prepared, 2) sections were deparaffinized through two changes of xylene, absolute, and 95% alcohols to distilled water, (3) staining was done in working crystal violet solution for one to two minutes, and, finally, (4) slides were rinsed well in tap water. This special stain should be seen immediately after preparation due to the lack of access to the special mount to paste the cover glass.

Congo red techniques of staining

Congo red solution was prepared with the dilution of 1-gr Congo red in 100-mL distilled water. Then, staining was performed on paraffin sections according to the following method: (1) an 8-µm section of the proper paraffin block was prepared, (2) sections were deparaffinized through two changes of xylene, absolute, and 95% alcohols to distilled water, (3) staining was done in Congo red solution 45 to 60 minutes, (4) the slides were dipped in the potassium hydroxide solution for 15 seconds, (5) The slides were washed in running water for 15 minutes, (6) the slides were counterstained lightly with hematoxylin for one to two minutes, (7) the slides were differentiated in 1% acid alcohol, (8) the slides were washed in water, (9) the slides were made blue in ammonia water, (10) the slides were washed in water, (11) the slides were dehydrated with two changes of 95% alcohol and absolute alcohol, cleared with two to three changes of xylene, and mounted.

Immunohistochemical techniques of staining 

IHC examination of HMWK and CK5 was performed on paraffin sections according to the following method., Sections (4 µm) of the proper paraffin block were prepared for each case, and the sections were covered with poly-lysine on a slide. Unstained slides were incubated in oven 60°C for 30 minutes. For deparaffinization, incubation in fur (T = 61-63°C for 20 minutes) was used. After that, the slides were placed in xylene (three times, each for 10 minutes) and gradually rehydrated by putting them in ethanol (100%, 96%, and 70% each for 30-45 seconds). Then, washing with phosphate buffer saline for five minutes was done. All slides were incubated in H2O2 3% for three minutes until no bubble was seen on the surface. After washing in phosphate buffer saline for five minutes, the antigen retrieval step was performed in TRIS buffer (1, 3-dichloro-2-propyl) (pH = 9) for 15 minutes. After this step, Dako Pen (Dako Denmark A/S, Glostrup, Denmark) was used, and diluted goat serum was added for 20 minutes (diluted by phosphate buffer saline to 10% concentration). In this phase, incubation of the slides was performed using optimally ready-to-use antibodies, HMWK (Clone 34betaE12, BioCare Medical, Pacheco, CA, USA), and CK5 (Clone XM26, Novocastra Laboratories Ltd., Newcastle upon Tyne, UK). Then, ready-to-use dilution was performed for an hour. After washing with phosphate buffer saline (two times, each for five minutes), EnVision (K5007, Dako Denmark A/S) was used, and the slides were washed with phosphate buffer saline (two times, each for five minutes). Then, DAB (3, 3’ diaminobenzidine) chromogen was added for five minutes after which washing with phosphate buffer saline (for five minutes) was done again. Finally, the slides were counterstained with hematoxylin, rinsed in running water for a few minutes, dehydrated in graded ethanol solutions, cleared with xylene, and mounted.

Evaluation of special stain slides

Crystal violet stain slides were evaluated for purplish violet amyloid deposits in the papillary dermis. Sections from medullary thyroid carcinoma were considered as positive controls for crystal violet stain. Congo red-stained slides were evaluated for apple-green birefringence of amyloid deposits under polarized light in the papillary and upper dermis.

 Evaluation of immunostained slides

Immunostained slides were evaluated using dermatopathologist for immunoreactivity in amyloid deposits. Glassy deposits, which were observed in the papillary dermis as a brown material or brown discoloration of collagen fibers in the upper dermis, were considered as a positive reaction. In each slide, the immunostaining of epidermal keratinocytes was considered as an internal positive control for HMWK and CK5.

Exclusion criteria

The specimens with inadequate tissue for preparation of all required sections and cases with only one 4-mm punch biopsy were excluded. In negative control cases, patients with MA in clinical or pathological differential diagnosis were excluded.

Statistical analysis

Data analysis was performed using SPSS Version 22 (IBM Corp., Armonk, NY, USA), chi-square test, and McNemar test. The comparison of H&E stained slides with Congo red stain, crystal violet stain, immunoreactivity of each marker, and the combination of both markers (HMWK and CK5) was performed.

## Results

In total, 42 cases with clinical differential diagnosis of MA and 14 cases with skin hyperpigmentation diagnosed through clinical differential other than MA as negative controls were included in this study. In the first group, 1 patient was male and 41 patients were female, resulting in a male-to-female ratio of 0.025. There was an age range of 19 to 55 years and a mean age of 35.5 ± 10.09 years. In the negative control group, 2 were males and 12 were females, resulting in a male-to-female ratio of 0.17. There was an age range of 5 to 52 years and a mean age of 26.5 ± 14.03 years. Upper back and arm were the most frequent sites of skin biopsies in cases with clinical differential diagnosis of MA followed by the chest, leg, flank, abdomen, and unknown sites (one patient). In the negative control group, the face was the most frequent site of skin biopsies with other sites including the trunk, back, leg, breast, axilla, buttock, and unknown sites (two patients). In this group, only two patients underwent two 4-mm punch biopsies from different sites in the involved area, while the others underwent one biopsy.

**Figure 1:**
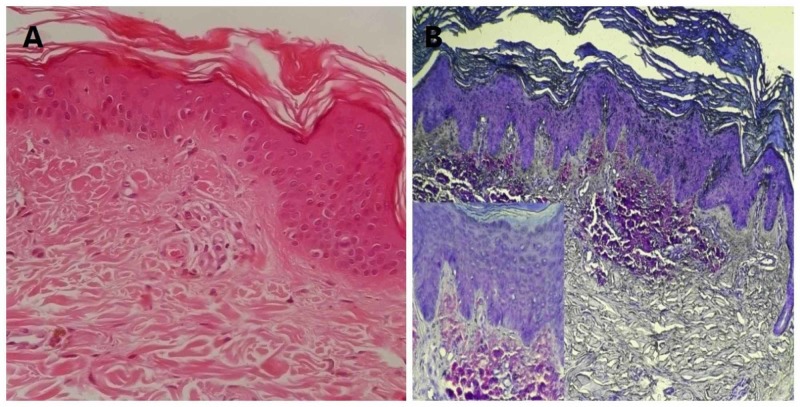
(A) Hyaline bodies in hematoxylin-eosin staining (200×). (B) Crystal violet staining of amyloid deposition in the papillary and upper dermis (200×). Inset shows purplish red amyloid deposits in the papillary and upper reticular dermis (crystal violet, 400×).

As expected, all control cases were negative for amyloid deposits in H&E, special stains, and IHC slides. In the case group, H&E-stained sections showed homogenization of papillary dermal collagen, pigment incontinence, apoptotic keratinocytes, and perivascular lymphocytic infiltration. Expansion of dermal papillae with globular deposits of eosinophilic, amorphous, and acellular hyaline bodies as a primary diagnostic clue was seen in 15 (35.7%) patients (Figure 1A). Congo red stain (Highman’s technique) did not show apple-green birefringence in the eosinophilic deposits in any of the cases. Evaluation of crystal violet stained sections showed purplish violet amyloid deposits in 15 (35.7%) cases (Figure 1B).

Immunohistochemistry using CK5 and HMWK displayed amyloid deposits in 22 (52.4%) and 21 (50%) patients, respectively. In this study, we have used two punch biopsies from two different sites in the involved area in an individual patient with clinical features of MA. Frequencies of positive and negative results in H&E, crystal violet, Congo red, and IHC in the first and second sites of biopsies are presented in Tables 1-3. According to Tables 1-3, the positivity of amyloid detection was not significantly different between single or double biopsy in any of the diagnostic techniques (p ≥ 0.05). 

**Table 1:** Frequencies of positive and negative results of hematoxylin-eosin staining in the first and second sites of biopsies and both sites

	First site	Second site	Both sites	p-Value
Positive	12 (28.6%)	12 (28.6%)	15 (35.7%)	0.22
Negative	30 (71.4%)	30 (71.4%)	27 (64.3%)	

**Table 2:** Frequencies of positive and negative results of crystal violet staining in the first and second sites of biopsies and both sites

	First site	Second site	Both sites	p-Value
Positive	13 (31%)	14 (33.3%)	15 (35.7%)	0.37
Negative	29 (69%)	28 (66.7%)	27 (64.3%)	

**Table 3:** Frequencies of CK5 and HMWK expression in the first and second sites of biopsies and both sites CK5, cytokeratin 5; HMWK, high molecular weight keratin

		First site	Second site	Both sites		p-Value
CK5	Positive	22 (52.4%)	19 (45.2%)	22 (52.4%)		0.05
	Negative	20 (47.6%)	23 (54.8%)	20 (47.6%)		
HMWK	Positive	18 (42.9%)	18 (42.9%)	21 (50%)		0.22
	Negative	24 (57.1%)	24 (57.1%)	21 (50%)		

 

**Figure 2:**
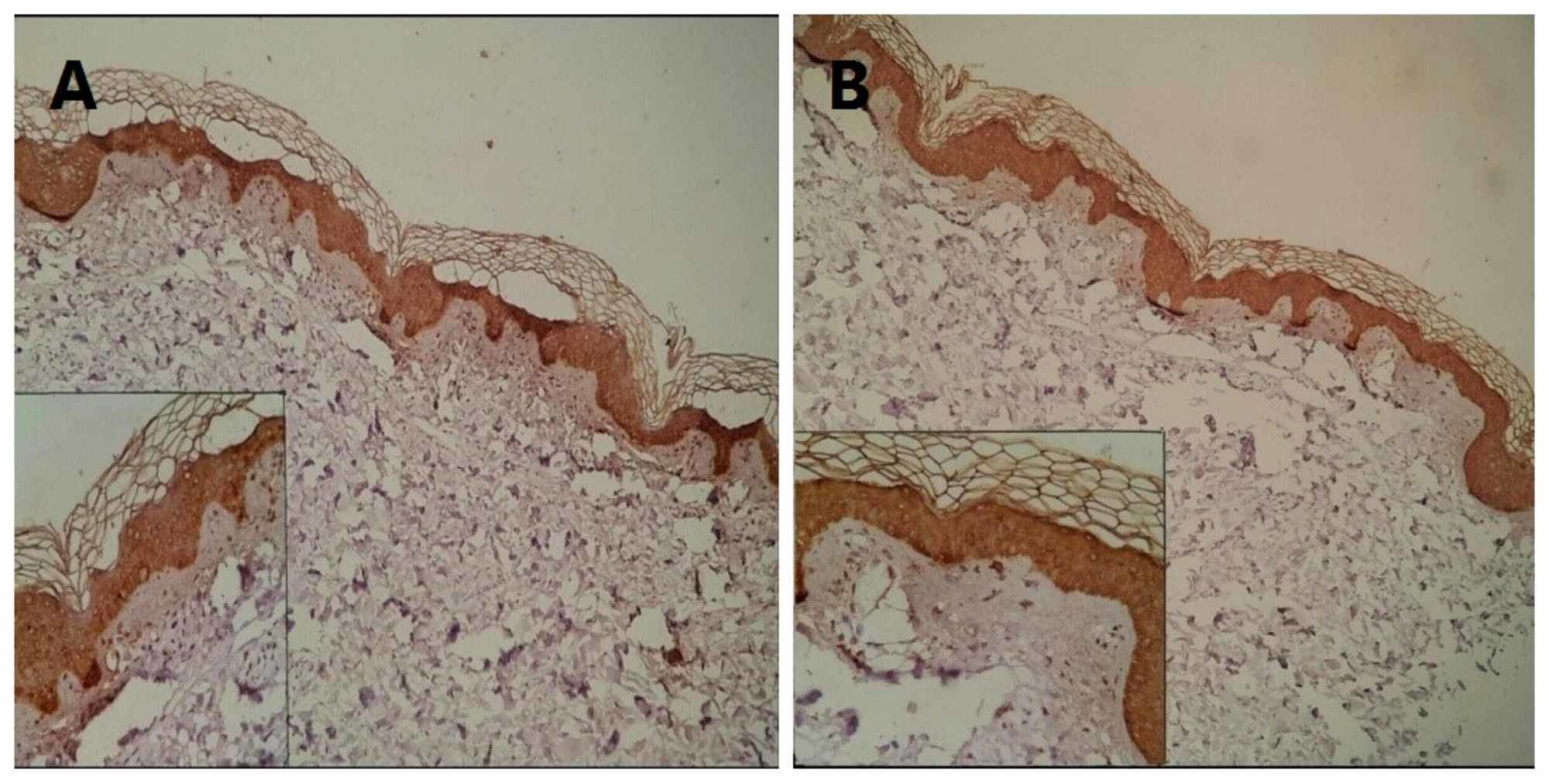
(A) CK5 immunostaining of keratinocytes (as internal control) and negativity for amyloid deposition (100×). (B) HMWK immunostaining of keratinocytes (as internal control) and negativity for amyloid deposition. Pigment incontinence is seen in the upper dermis (100×). Inset (A, B) shows pigment incontinence and negativity for amyloid deposition (200×). CK5, cytokeratin 5; HMWK, high molecular weight keratin

Positivity in IHC markers after the evaluation of negative control slides was determined. According to these examinations, very mild discoloration of collagen fibers and very scanty depositions of brown color materials in a blue background were considered as negative results (Figure 2).

**Figure 3:**
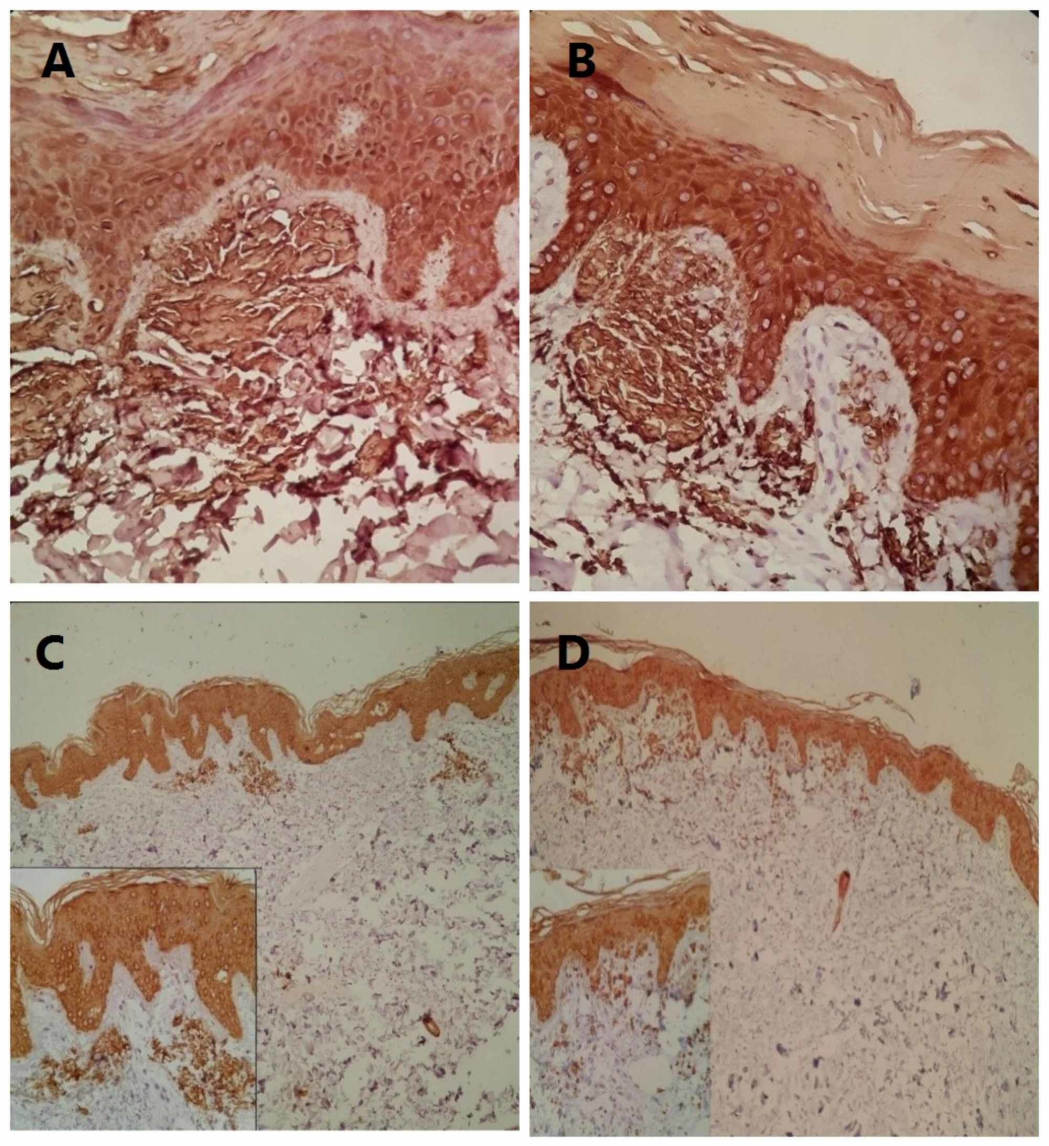
(A) CK5 staining of amyloid deposition in the papillary dermis (200×). (B) HMWK staining of bulky amyloid deposition in the papillary dermis (200×). (C) CK5 staining with patchy immunoreactivity for amyloid depositions in the upper dermis (100×). Inset shows discrete amyloid deposit in the upper dermis (200×). (D) HMWK staining with focal immunoreactivity for amyloid depositions in the papillary and upper dermis (100). Inset shows amyloid deposit in the papillary dermis (200×). CK5, cytokeratin 5; HMWK, high molecular weight keratin

**Figure 4:**
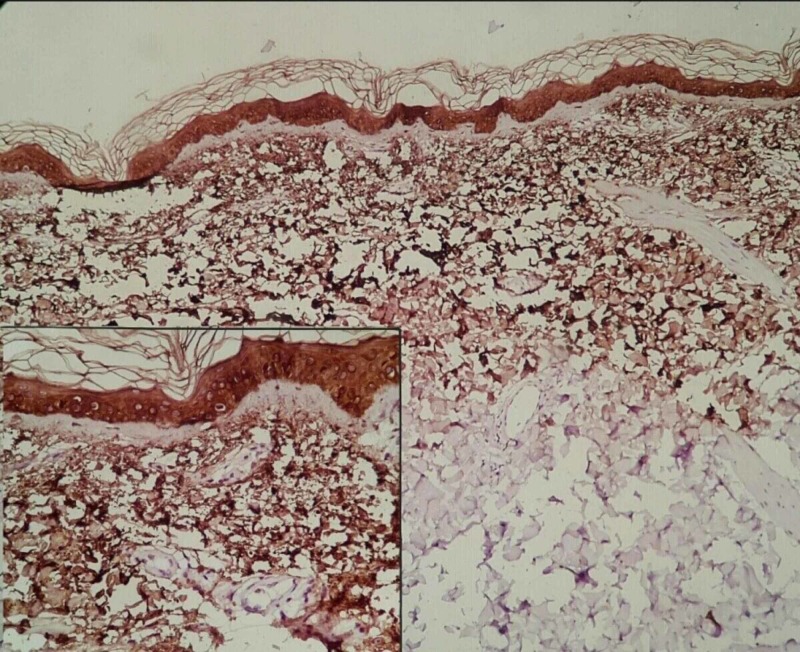
CK5 staining with diffuse immunoreactivity for amyloid deposition in the upper dermis in a granular, fibrillary pattern (on collagen fibers) (100×). Inset shows amyloid deposit in the upper dermis (200×). CK5, cytokeratin 5

Pigment incontinence in IHC staining presents a brown color granular deposition in the cytoplasm of the histiocytes in the papillary dermis, which should not be misdiagnosed as amyloid deposition. Different patterns of positive immunoreactivity of CK5 and HMWK are shown in Figures 3 and 4.

 

Tables 4-7 summarize the comparison of H&E staining, crystal violet staining, and IHC expression of CK5 and HMWK. H&E staining did not have a significant difference with crystal violet (p = 1) (Table 4).

**Table 4:** Comparison of H&E stain with crystal violet stain H&E, hematoxylin-eosin

		Crystal violet		
		Positive	Negative	Total
H&Estain	Positive	11 (26.2%)	4 (9.52%)	15 (35.7%)
	Negative	4 (9.52%)	23 (54.76%)	27 (64.3%)
	Total	15 (35.7%)	27 (64.3%)	42 (100%)
	P-value	1		

Immunohistochemistry using either CK5 or HMWK improved the detection of amyloid deposit as compared with H&E (52% and 50% versus 37.5%; p ˃ 0.05), whereas they showed a significant difference with using both IHC markers (p = 0.039) (Table 5).

**Table 5:** Comparison of H&E stain with immunohistochemical expression of CK5, HMWK, and both markers CK5, cytokeratin 5; HMWK, high molecular weight keratin, H&E, hematoxylin-eosin

		CK5		HMWK		Both markers	
		Positive	Negative	Positive	Negative	Positive	Negative
H&Estain	Positive	13 (30.95%)	2 (4.76%)	13 (30.95%)	2 (4.76%)	13 (30.95%)	2 (4.76%)
	Negative	9 (21.43%)	18 (42.86%)	8 (19.05%)	19 (45.24%)	10 (23.81%)	17 (40.47%)
	Total	22 (52.4%)	20 (47.6%)	21 (50%)	21 (50%)	23 (54.76%)	19 (45.24%)
	P-value	0.065		0.109		0.039	

As shown in Table 6, IHC stain using CK5, HMWK, or both markers has a significant difference with crystal violet (p = 0.008).

**Table 6:** Comparison of crystal violet stain with IHC expression of cytokeratin 5, HMWK, and both IHC markers CK5, cytokeratin 5; HMWK, high molecular weight keratin; IHC, immunohistochemical

		CK5		HMWK		Both markers	
		Positive	Negative	Positive	Negative	Positive	Negative
Crystal violet	Positive	15 (35.71%)	0 (0%)	15 (35.71%)	0 (0%)	15 (35.71%)	0 (0%)
	Negative	7 (16.67%)	20 (47.62%)	6 (14.29%)	21 (50%)	8 (19.05%)	19 (45.24%)
	Total	22 (52.38%)	20 (47.62%)	21 (50%)	21 (50%)	23 (54.76%)	19 (45.24%)
	P-value	0.016		0.031		0.008	

As shown in Table 7, using both IHC markers is not significantly more diagnostic than each of them (p > 0.05).

**Table 7:** Comparison of IHC expression of CK5 and HMWK CK5, cytokeratin 5; HMWK, high molecular weight keratin; IHC, immunohistochemical

		CK5		HMWK	
		Positive	Negative	Positive	Negative
Both IHC markers	Positive	22 (52.38%)	1 (2.38%)	21 (50%)	2 (4.76%)
	Negative	0 (0%)	19 (45.24%)	0 (0%)	19 (45.24%)
	Total	22 (52.38%)	20(47.62%)	21 (50%)	21 (50%)
	P-value	1		0.5	

In the examination of Congo red stained slides, none of the patients showed apple-green birefringence under polarized light, whereas positive controls (medullary thyroid carcinoma tissue sections) were positive. Therefore, tables related to this special stain and its comparison with other stains were not presented in this study.

## Discussion

MA is a rare subtype of PCA whose definite diagnosis depends on special stains or IHC. Even an expert dermatopathologist may obtain only a primary clue by examination of H&E slides. Some studies have evaluated special stains and IHC markers for the diagnosis of amyloid deposition in the skin in different types of amyloidosis. Previous studies on this subtype have used a few cases; however, such studies are rare.

The mean age of our cases was 35.5 ± 10.9 years, with female preponderance, which is similar to most previous studies [11-14]. The results on H&E examination showed the globular deposition of hyaline bodies in papillary dermis in 15 out of 42 patients, which is less than the results obtained by Layegh et al., who observed hyaline globules in 28 out of 54 patients [14]. This discrepancy may be due to the fact that the author evaluated both subtypes of macular and lichen amyloidosis.

Special stains used in different studies had contradictory results. Vijaya et al. examined 32 cases with PCA, 7 with MA, and 25 with lichen amyloidosis [15]. All of the patients showed apple-green birefringence under polarized light. Abdullah Alhumid and Abdulgader Fathaddin examined 16 cases of MA and 3 cases of lichen amyloidosis, in which only 1 case showed apple-green birefringence under polarized light [11]. Patterson used the crystal violet stain to confirm the amyloid deposits, which is superior to both alkaline and Highman’s Cong red stains [16]. In our study, none of the patients showed apple-green birefringence under polarized light. In this study, we observed a similar diagnostic rate in crystal violet and H&E staining for a dermatopathologist (35.7% in each method). Previous studies evaluating crystal violet staining in the diagnosis of MA are rare.

A few IHC markers have been reported to be helpful in the diagnosis of MA. CK5, CK5/6, CK18, and HMWK (34betaE12) are positive in amyloid deposits [3,11,16]. We found a rate of 50% positivity using HMWK and 52.4% using CK5, another study reported the positivity of these two markers in 100% of the patients with PCA [17]. Positivity of CK5/6 in 16 (100%) out of 16 patients with MA was reported in another study [11]. Apaydin et al. reported a positivity of CK5/6/18 in 6 out of 12 and positivity of CK1-8 (AE3) in 4 out of 12 MA cases [9]. These differences between the studies may be explained using different clones or types of cytokeratins and different qualities of IHC markers.

Our series of comparisons using CK5 and HMWK did not display significant differences, which agrees with the study by Chang et al. [17]. Also, the findings using CK5 or HMWK did not display significant differences with H&E stain, whereas using both IHC markers had a significant difference (p = 0.039). The findings using each of the IHC markers had a significant difference with crystal violet stain (52.4% and 50% with CK5 and HMWK, respectively, versus 35.7% with crystal violet; p = 0.008). In previous studies, such a comparison has not been made and therefore a comparison with our results could not be made.

Our results using two punch biopsies from two different sites in the involved area in comparison with using one of them did not display a significant difference in any of the staining methods (p > 0.05). The previous studies did not compare double and single biopsies for the diagnosis of MA.

Electron microscopic tissue study is the golden standard for the detection of amyloid deposits in MA; therefore, we suggest that at least some positive and negative cases be examined through this method in order to compare the results with each of the stainings. Since the calculation of sensitivity and specificity of the IHC markers are required, evaluation of at least 200 samples is suggested in future studies. Collecting the results of both studies determines the sensitivity and specificity of each marker.

## Conclusions

Based on the results, Congo red stain does not seem helpful in the diagnosis of MA. Crystal violet stain is not more sensitive than H&E stain. Therefore, we suggest that IHC staining might be a useful tool for diagnostic purposes in all cases with a clinical differential diagnosis of MA. We therefore recommend the IHC profile that includes CK5 and HMWK (34betaE12). There is no need for the use of two punch biopsies in diagnosis.
